# Prenatal depression exposure alters white matter integrity and neurodevelopment in early childhood

**DOI:** 10.1007/s11682-021-00616-3

**Published:** 2022-01-09

**Authors:** Annerine Roos, Catherine J. Wedderburn, Jean-Paul Fouche, Shantanu H Joshi, Katherine L Narr, Roger P Woods, Heather J Zar, Dan J. Stein, Kirsten A. Donald

**Affiliations:** 1 Department of Psychiatry, Stellenbosch University, Cape Town, South Africa; 2 Department of Pediatrics and Child Health & SAMRC Unit on Child and Adolescent Health, University of Cape Town, Cape Town, South Africa; 3 Neuroscience Institute, University of Cape Town, Cape Town, South Africa; 4 Department of Clinical Research, London School of Hygiene & Tropical Medicine, London, UK; 5 Department of Psychiatry and Mental Health, University of Cape Town, Cape Town, South Africa; 6 Department of Neurology and Bioengineering, University of California, Los Angeles, CA USA; 7 Department of Neurology and of Psychiatry and Biobehavioral Sciences, University of California, Los Angeles, CA USA

**Keywords:** prenatal exposure, maternal depression, white mater integrity, development

## Abstract

Prenatal exposure to maternal depression increases the risk for onset of emotional and behavioral disorders in children. We investigated the effects of exposure to prenatal depression on white matter microstructural integrity at birth and at 2-3 years, and associated neurodevelopment. Diffusion-weighted images were acquired for children of the Drakenstein Child Health Study at 2-4 weeks postpartum (n=70, 47% boys) and at 2-3 years of age (n=60, 58% boys). Tract-Based Spatial Statistics was used to compare, using an ROI based approach, diffusion tensor metrics across groups defined by presence (>19 on Beck’s Depression Inventory and/or >12 on the Edinburgh Postnatal Depression Scale) or absence (below depression thresholds) of depression, and associations with neurodevelopmental measures at age 2-3 years were determined. We did not detect group differences in white matter integrity at neonatal age, but at 2-3 years, children in the exposed group demonstrated higher fractional anisotropy, and lower mean and radial diffusivity in association tracts compared to controls. This was notable in the sagittal stratum (radial diffusivity: p<0.01). Altered white matter integrity metrics were also observed in projection tracts, including the corona radiata, which associated with cognitive and motor outcomes in exposed 2-3-year-olds (p<0.05). Our findings of widespread white matter alterations in 2-3-year-old children with prenatal exposure to depression are consistent with previous findings, as well as with neuroimaging findings in adults with major depression. Further, we identified novel associations of altered white matter integrity with cognitive development in depression-exposed children, suggesting that these neuroimaging findings may have early functional impact.

## Introduction

Depression remains a prevalent and debilitating condition, affecting 264 million of the world’s population of all ages (Depression, 2020). The burden of perinatal depression is higher in low-to-middle income countries (LMIC) where multiple risk factors impact on quality of life (Pellowski et al., 2019; Woody et al., 2017). The rates of prenatal depression are as high as 47% in South African women in particular (Manikkam & Burns, 2012; Rochat et al., 2011). An emerging literature suggests that prenatal exposure to maternal psychosocial distress, which includes depression, may impact the development of brain networks in exposed children, with associated increased risk for later behavioral and mental disorders (Scheinost et al., 2017; Van Den Bergh et al., 2018). Maternal depression during pregnancy (PDE), specifically, has a reported association with emotional and behavioral disorders in children (Giallo et al., 2015; O’Donnell et al., 2014). PDE is believed to exert its effects, in part, through biological mechanisms that include higher circulating glucocorticoid levels in the developing fetus, resulting in reprogramming of the hypothalamic-pituitary-adrenal (HPA) axis during a critical developmental window (Fatima et al., 2017). Research has recently started to identify relationships between maternal mood during pregnancy, fetal physiological programming, including alterations in both HPA axis and immune system, and later child psychopathology (Glover et al., 2018; Monk et al., 2019). Likewise, PDE has also been reported to impact early general physical health in exposed children, predicting subsequent health-related stress, depression, and impaired social functioning at age 20 years (Raposa et al., 2014).

Neuroimaging studies suggest that altered brain structure is observable even in very young children following PDE. Diffusion tensor imaging (DTI) studies in infants with PDE have reported differences in white matter microstructural integrity of frontal and limbic regions compared to unexposed control infants (Dean et al., 2018; Graham et al., 2020; Rifkin-Graboi et al., 2013), although not all data are consistent (Jha et al., 2016). In older children (spanning 2-10 years) differences in white matter integrity have been observed in frontal, temporal and limbic regions (El Marroun et al., 2018; Hay et al., 2020; Lebel et al., 2016). Further, altered white matter integrity of frontal-limbic networks in toddlers with PDE has been shown to predict internalizing and externalizing behaviors (Hay et al., 2020; Wee et al., 2017).

Children born in LMICs in particular may be exposed to a range of other environmental and psychosocial risk, putting them at higher risk of lost developmental potential. Differentiating the impact of these additional prenatal exposures, including maternal alcohol and tobacco smoking, often highly prevalent in depressed pregnant women of lower socio-economic environments (Vythilingum et al., 2012), remains difficult. Prenatal alcohol exposure may alter HPA axis function in exposed children (Franks et al., 2020), and has shown widespread changes in white matter microstructure (Ghazi Sherbaf et al., 2019). Prenatal tobacco exposure also impacts brain structure and behavior in young exposed children (Ekblad et al., 2010; El Marroun et al., 2016). The aim of this study is to determine the effects of PDE on white matter microstructural integrity in children followed from birth to 2-3 years of age, in the Drakenstein Child Health Study (DCHS) birth cohort situated in a LMIC setting, and to examine associations with neurodevelopmental markers. Based on putative involvement of frontal, temporal and limbic regions in children with PDE, we expected to see distributed alterations in white matter integrity. As noted above, since alcohol use and tobacco smoking may also influence brain maturation, these factors were included as potential confounders when investigating the effects of PDE.

## Methods

### Study design and participants

**Fig. 1:**
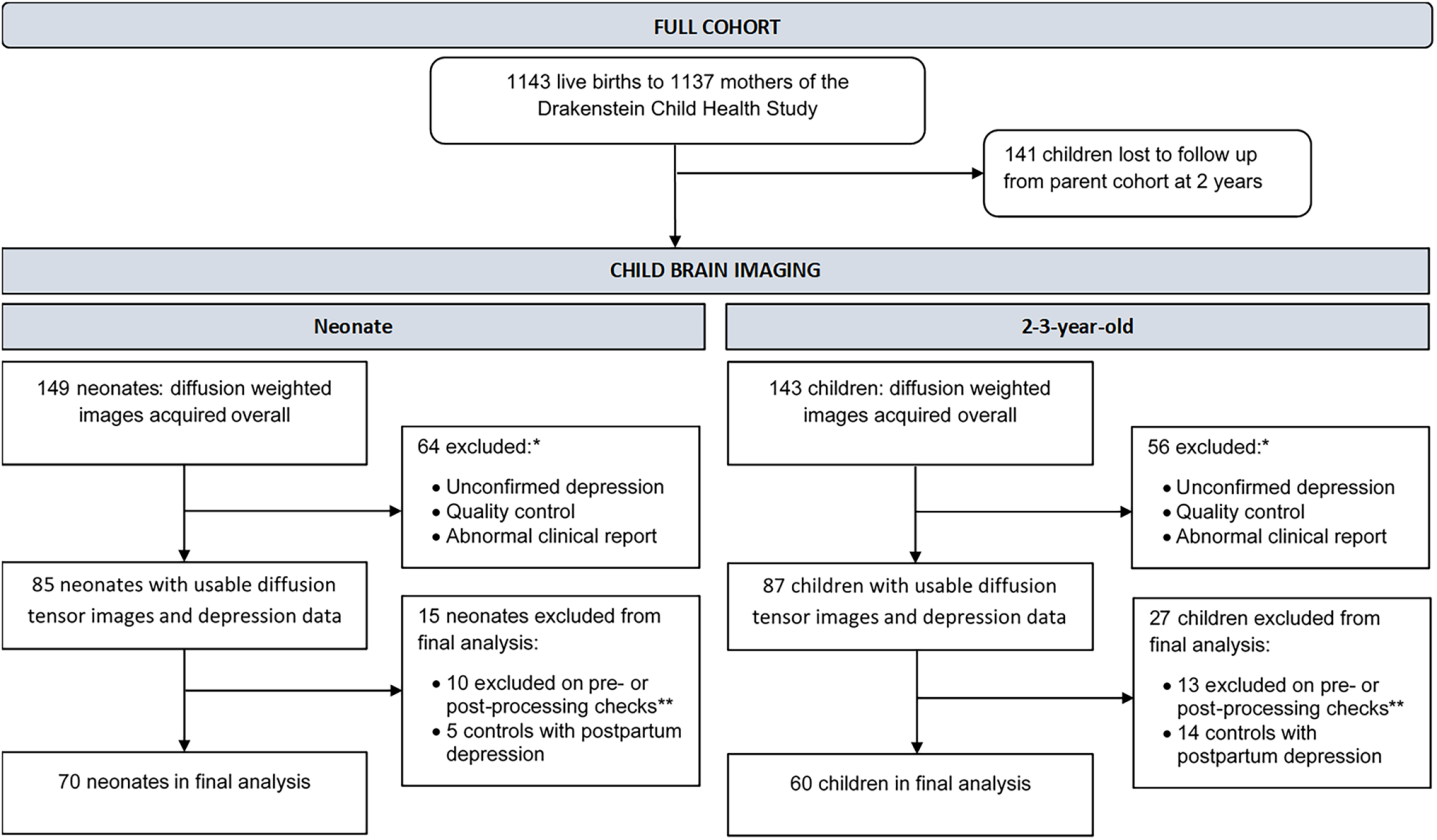
Flowchart of participation in the main Drakenstein Child Health Study and neuroimaging sub-study. DTI data of 70 neonates and 60 toddlers were available for full image processing and statistical analyses. * Depression was unconfirmed when not assessed. Reasons for exclusion due to quality control: obvious motion, technical or other artefacts; either AP or PA unusable. The same two participants were excluded in both the neonatal and 2 year analyses for an abnormal clinical report. ** Participant data exclusions: prematurity, maternal illicit substance use, and failing preprocessing in the final analysis

This study is a population-based cohort study investigating early determinants of child health and development. The sample represent a sub-group of the DCHS birth cohort situated in a peri-urban region in South Africa (Stein et al., 2015; Zar et al., 2015). Pregnant women aged ≥18 years were recruited during their second trimester while attending routine antenatal care. Mothers were approached after delivery for potential inclusion of their children in the neuroimaging study. Children were excluded from this sub-study if the mother had a positive urine screen for substance use other than alcohol or tobacco (e.g. methamphetamine, cocaine); if they were <36 weeks gestation; had an Apgar score of <7 at 5 minutes; major neonatal complications, an identified genetic or congenital syndrome; or standard contraindications to MRI. See Fig. 1 for a flow chart of participation in the DCHS.

Mothers provided informed written consent for themselves and their child’s participation in both the main DCHS and the neuroimaging component. The study was approved by the Health Research Ethics Committee of the University of Cape Town (UCT HREC: 401/2009, 525/2012), and conducted according to the principles of the Declaration of Helsinki.

### Maternal assessment

Mothers completed demographic and psychosocial assessments during trimester two, including reporting on depressive symptoms and substance use. Detailed descriptions of all assessments on the cohort are provided elsewhere (Donald et al., 2018; Stein et al., 2015). Children of mothers who had a score of >19 on the Beck’s Depression Inventory (BDI) and/or >12 on the Edinburgh Postnatal Depression Scale (EPDS) prenatally, representing moderate to severe depression symptomatology, were included in the PDE group. The BDI is a 21-item scale that evaluates depression symptoms per item on a scale of 0 (no symptoms) to 3 (severe symptoms). In similar fashion, the EPDS assesses depression in the past 7 days using 10 items. On both scales, a higher score indicates more severe depression. These scales are widely validated to assess prenatal or postnatal depression (Beck et al., 1988; Cox et al., 1987; Tsai et al., 2013). The whole cohort prevalence of maternal depression during pregnancy was 28% (Stein et al., 2015). Imaged children of mothers with low or no depression, thus scoring below the threshold on these scales during both pre- and postnatal periods were included in the control group. Additional substance exposure was defined for alcohol exposure as mothers who scored >11 on the Alcohol, Smoking and Substance Involvement Screening Test (ASSIST), and active maternal tobacco smoking as maternal urine cotinine levels >500 ng/ml in the second trimester. The ASSIST is validated by the World Health Organization for use in this context (Humeniuk et al., 2008; Jackson et al., 2010).

### Child imaging

Neuroimaging of children was performed at 2-4 weeks, and later, at 2-3 years of age, during natural sleep as described previously (Wedderburn et al., 2020). Children were monitored in the scanner room by a qualified team member throughout the duration of the scan. Anthropometric measurements were taken at the neuroimaging visit.

Imaging protocols were age-appropriate, thus set up per time point to be optimal for developmental stage. T2 structural images which provide superior contrast were acquired at the neonatal age using a 3T Siemens Allegra MRI scanner and single-channel head coil. T2-weighted structural images had the following parameters: TR=3500 ms; TE=354 ms; 128 slices; voxel size 1.0×1.0x1.0 mm; FOV 160x160x128 mm; slice thickness 1.0 mm; scan duration 5 min 41 s. Two diffusion-weighted images were acquired in an anterior-posterior and posterior-anterior encoding direction, with the following parameters: 45 non-collinear gradient directions; one b-value of 0 and of a 1000 s/mm^2^; TR=7900 ms; TE=90 ms; voxel size 1.3x1.3x1.6 mm^3^; FOV 160x160x97 mm; slice thickness 1.6 mm; scan duration per encoding direction of 6 min 27 s.

At the 2-3 year time point T1-weighted structural images were acquired using a 3T Siemens Skyra MRI scanner and 32-channel head coil optimized for young children. Parameters were: TR=2530ms; TE(1-4)=1.69, 3.54, 5.39, 7.24; flip angle = 7°; voxel size: 1.0×1.0×1.0 mm; FOV 224x224x176 mm; slice thickness 1.0mm; scan duration 5 min 21s. Two diffusion-weighted images were also acquired in an anterior-posterior and posterior-anterior direction, with the following parameters: 30 diffusion directions; one b-value of 0s/mm^2^ and of a 1000s/mm^2^; TR 7800 ms; TE 92 ms; voxel size: 1.8×1.8×2.0 mm; FOV 230x230x121 mm; slice thickness 2 mm; scan duration per encoding direction of 8 min 36 s.

### Neurodevelopmental assessment

Neurodevelopment of the child was assessed at 24 months using the Bayley Scales of Infant and Toddler Development (BSID-III) (Bayley, 2006) as described previously (Donald et al., 2019). Domains assessed were motor skills, language, cognition, social-emotional and adaptive functioning. The Child Behavior Checklist (CBCL) was used to assess internalizing and externalizing behavior at 24 months (Achenbach & R. L. A., 2000).

### Image processing

Diffusion data was preprocessed using TORTOISE (Tolerably Obsessive Registration and Tensor Optimization Indolent Software Ensemble) (Irfanoglu et al., 2017; Pierpaoli et al., 2010), on the Centre for High Performance Computing cluster (CHPC, Cape Town). TORTOISE implements comprehensive correction and applies greater anatomical registration ability compared to mainstream diffusion processing pipelines, optimizing results for pediatric populations who are inclined to move (Taylor et al., 2016).

Raw diffusion-weighted images were quality checked for movement and technical artefacts before preprocessing, to ensure that at least 15 volumes per image were usable. Individual structural images served as anatomical reference. In the neonate, T2 images were registered to the University of North Carolina (UNC) neonate structural template (Shi et al., 2011) given its relative immature tract formation compared to the 2-3-year brain that has core structure in place. The registered neonate image was then used as a reference image in TORTOISE. In the 2-3-year-olds, T1-images were inverted to have similar contrast to the diffusion b0 volume. Distortion corrections for participant motion, eddy currents and basic echo-planar imaging (EPI) distortions were computed separately on each anterior-posterior and posterior-anterior encoded image, using the DIFF PREP module in TORTOISE Gradient directions were rotated and adjusted according to the eddy current and motion correction. Encoded sets were merged and further EPI distortion corrections done using the DR BUDDI module (Irfanoglu et al., 2015).

The Tract-based Spatial Statistics (TBSS) pipeline in FMRIB Software Library (FSL version 5) (Smith et al., 2006) was used to fit tensors and extract diffusion parameters from preprocessed diffusion images using an ROI approach. Parameters included fractional anisotropy (FA), and measures of different aspects of diffusivity including mean diffusion (MD) that represents the average trace diffusivity, radial diffusion (RD) representing diffusion perpendicular to the tensor and axial diffusion (AD) representing diffusion aligned with the tensor. A representative template was created during the registration step of TBSS, utilizing images from our sample. This approach has been recommended in research where brains that are likely to differ from an adult MNI template are being investigated and has also been suggested for studies with smaller sample sizes such as ours. The recommended approach allows enhanced registration quality and thereby optimizes results (Bach et al., 2014; Smith et al., 2006).

Following preprocessing with TORTOISE, FSL was used to create FA images. During this process BET did brain extraction and DTIFIT tensor extraction (Smith, 2002). The first step of TBSS was then initiated to prepare images. Secondly, the representative template was created using individual images from our cohort. This template was registered to FSL’s standard FMRIB58_FA image as an intermediate step, and individual images were linearly registered into standard MNI space using FLIRT (Jenkinson et al., 2002) by applying the transforms calculated by registering the template to the FMRIB58_FA image. All participant images were then merged into an average FA image for use as the registration target. Thirdly, TBSS created a mean FA image and skeleton. Finally, this mean FA skeleton was thresholded at a level of 0.15. This step created masks delineating image voxels, and a distance map to enable voxel-wise projection of single FA images onto the mean FA skeleton. After skeletonization, ROI masks of the mean FA skeleton were then created for all 48 white matter tract regions as per the JHU ICBM-DTI-81 atlas (Mori et al., 2008), and the mean DTI metrics for each of the 48 regions were extracted from template space, using standard TBSS procedures, for further statistical analysis. MD, RD and AD images were also derived from the mean FA skeleton and ROIs extracted for the 48 regions for each of these parameters.

### Statistical analysis

We investigated group differences (PDE, control) for each age cohort (neonates, or 2-3 year olds) individually, thus dealt with each time point using a cross-sectional approach due to different white matter developmental stages. For these analyses diffusion parameters across tracts i.e. ROIs were examined between groups using separate general linear models firstly controlled for sex and age at scanning, and secondly sex, age at scanning, alcohol use and active tobacco smoking in Statistica version 13. Sex and age were selected *a priori* due to known biological effects on white matter diffusion (Geng et al., 2012; Long et al., 2018; Paolozza et al., 2017). Further, multiple linear regression modeling was used to investigate associations within groups of each neurodevelopmental domain with tracts showing significant group differences. Tracts were categorized by type, in accordance with the JHU white matter atlas derived from stereotaxic anatomical classification by Mori et al. (2008), given potential functional significance at different stages of neurodevelopment (Roos et al., 2021). These include association (sagittal stratum, external capsule, cingulum, fornix, superior longitudinal fasciculus, superior fronto-occipital fasciculus, uncinate fasciculus), brain stem (cerebellar peduncle, medial lemniscus, corticospinal tract), commissural (corpus callosum, tapetum), projection (cerebral peduncle, internal capsule, pontine crossing, corona radiata, posterior thalamic radiation) and limbic tracts (fornix, fornix stria terminalis, uncinate fasciculus) (Mori et al., 2008). Given a priori expectations about more diffuse group effects, partial eta squared values are presented as an indication of effect size (Cohen, 1969; Richardson, 2011). False Discovery Rate (FDR) correction for multiple comparisons was applied post hoc by tract type.

## Results

The mean age of the neonates was 3 weeks (range 2-5 weeks), and 2-3-year-olds were 34 months (range 30-37 months). Detailed demographic details are shown in Table 1. Although of the same birth cohort, samples overlapped partly between the two time points given the challenges of imaging young children associated with movement artefacts, and loss to follow-up. Weights of children in the two groups were similar at each time point. However, at neonatal age, head circumference and height were significantly lower in the PDE group compared to controls (p<0.05). Maternal alcohol use and tobacco smoking were significantly higher in the PDE group compared to controls (p<0.05).

**Table 1:** Demographic and clinical detail of participants

	Neonate			2-3-Year-old		
	PDE (n=37)	Control (n=33)		PDE (n=24)	Control (n=36)	
Mother	mean (SD) / n (%)		p	mean (SD) / n (%)		p
BDI	24.32 (9.80)	5.80 (5.73)	<0.001	25.08 (10.51)	3.17 (5.05)	<0.001
EPDS	13.84 (4.92)	7.17 (2.95)	<0.001	13.63 (5.76)	8.09 (3.09)	<0.001
Education			0.017			0.301
Complete secondary to any tertiary	13 (35%)	21 (64%)		9 (38%)	9 (25%)	
Primary to some secondary	24 (65%)	12 (36%)		15 (62%)	27 (75%)	
Alcohol use	10 (27%)	0 (0%)	0.001	6 (25%)	0 (0%)	0.002
Tobacco smoking	19 (51%)	8 (24%)	0.020	12 (50%)	9 (25%)	0.034
HIV infection	3 (8%)	11 (33%)	0.008	2 (8%)	15 (42%)	0.004
Child						
Gestation, weeks	38.95 (1.90)	39.27 (1.35)	0.415	39.25 (2.07)	39.56 (1.56)	0.517
Sex, boys	15 (41%)	17 (52%)	0.358	11 (46%)	25 (69%)	0.067
Age, days / months	21.32 (6.44)	21.49 (5.46)	0.911	34.35 (1.88)	34.10 (1.39)	0.561
Weight, kg	3.09 (0.50)	3.17 (0.42)	0.460	13.47 (1.99)	13.67 (1.45)	0.652
Head circumference, cm	35.70 (1.53)	36.88 (1.42)	0.002	49.27 (1.85)	49.89 (1.30)	0.131
Height, cm	49.35 (3.52)	51.06 (3.07)	0.035	91.50 (3.93)	91.29 (3.24)	0.838

### Group differences in white matter integrity

At neonatal age, there were no group differences in white matter integrity. Controlling for maternal education as an indicator of socio-economic status, which was significantly different between groups only at this time point (p = 0.017), did not alter results when the analysis was repeated in post-hoc analyses.

**Fig. 2:**
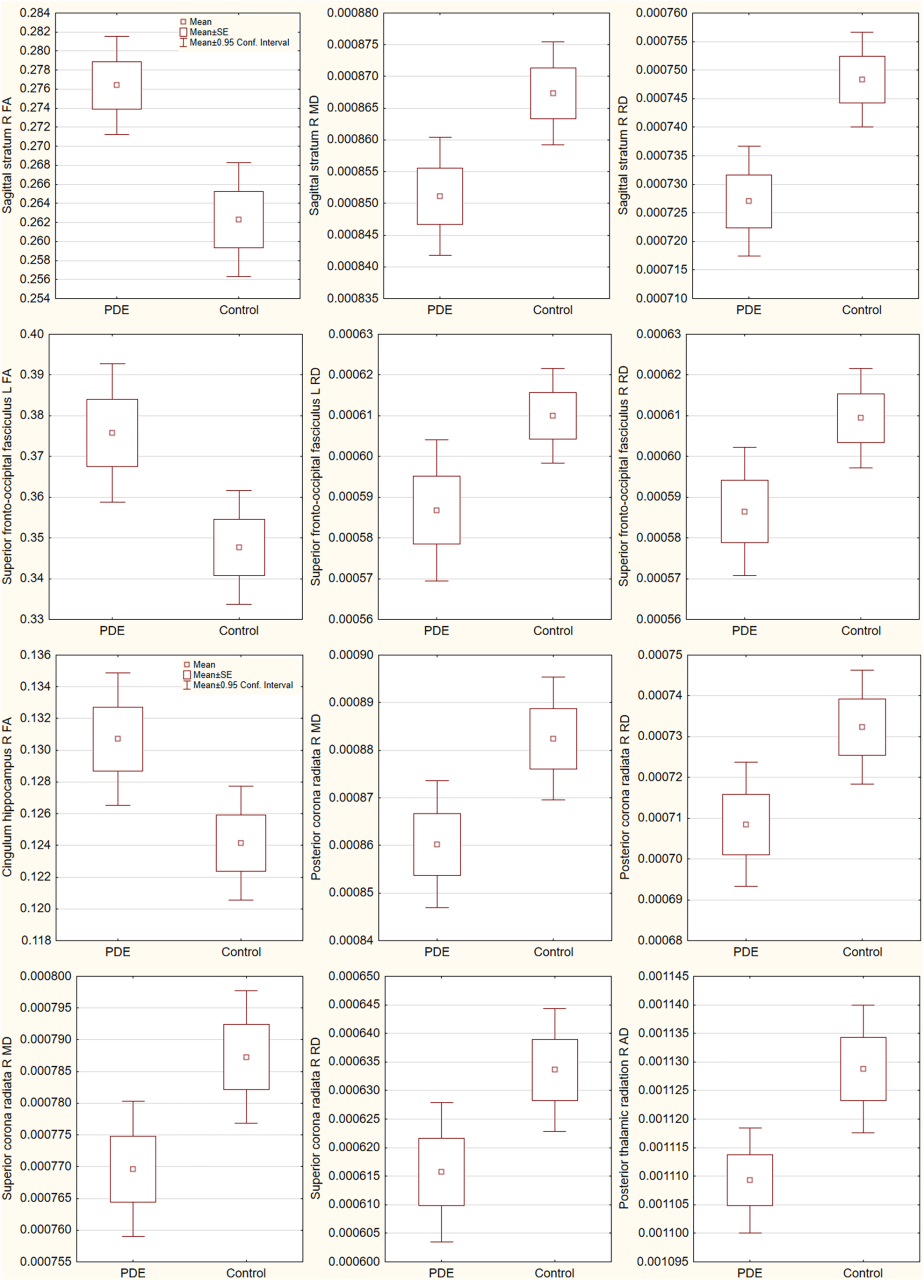
Boxplots of group effects associated with prenatal depression exposure (PDE) in 2-3-year-olds. Fractional anisotropy (FA) was significantly increased, and mean (MD), radial (RD) and axial (AD) diffusivity decreased in the PDE group compared to control children. As indicated in the top left graph, boxplots denote the mean parameter value with indication of the standard error (SE) and 0.95 confidence interval from the mean

At age 2-3 years, group differences in DTI metrics are presented in Table 2 and Fig. 2. FA was significantly increased, and MD, RD and AD decreased in the PDE group compared to unexposed controls in a number of tracts, controlling for sex and age at scanning. The differences were observed in association tracts (sagittal stratum, superior frontal-occipital fasciculus, hippocampal portion of the cingulum); and projection tracts (corona radiata, posterior thalamic radiation) with medium to large effect sizes. Group differences in RD in the right sagittal stratum survived FDR correction (corrected p = 0.022; controlling for sex, and age at scanning). After additionally adjusting for alcohol use and tobacco smoking in the general linear models, some group effects were reduced in significance, but effects remained notable for the sagittal stratum (increased FA and decreased RD with p<0.01) with medium to large effect sizes, without surviving correction.

**Table 2:** Group differences in white matter integrity at age 2-3 years

Region	Tract type	Effect in PDE	^1^Group		^2^Group	
			Partial eta^2^	p	Partial eta^2^	p
Sagittal stratum R	Association	↑ FA	0.1423***	0.004	0.1359**	0.006
		↓ MD	0.1168**	0.009	0.0927**	0.024
		↓ RD	0.1702***	0.001	0.1506***	0.003
Superior fronto-occipital L	Association	↑ FA	0.1227**	0.007	0.0818**	0.034
		↓ RD	0.0870**	0.025	0.0267*	0.233
Superior fronto-occipital R	Association	↓ RD	0.0775**	0.034	0.0258*	0.241
Cingulum hippocampal R	Association/limbic	↑ FA	0.1006**	0.015	0.0807**	0.036
Posterior corona radiata R	Projection	↓ MD	0.0882**	0.024	0.0676**	0.055
		↓ RD	0.0926**	0.020	0.0783**	0.039
Superior corona radiata R	Projection	↓ MD	0.0817**	0.030	0.0411*	0.138
		↓ RD	0.0731**	0.040	0.0373*	0.158
Posterior thalamic radiation R	Projection	↓ AD	0.0801**	0.031	0.0616**	0.068

1: Model including sex, and age at scanning.

2: Model including sex, age at scanning, alcohol use and tobacco smoking.

A small, medium and large effect size respectively correspond to values of 0.0099, 0.0588, and 0.1379 (Cohen, 1969).

***large effect size

**medium effect size

*small effect size

### Associations of white matter integrity with neurodevelopmental outcomes

**Fig. 3:**
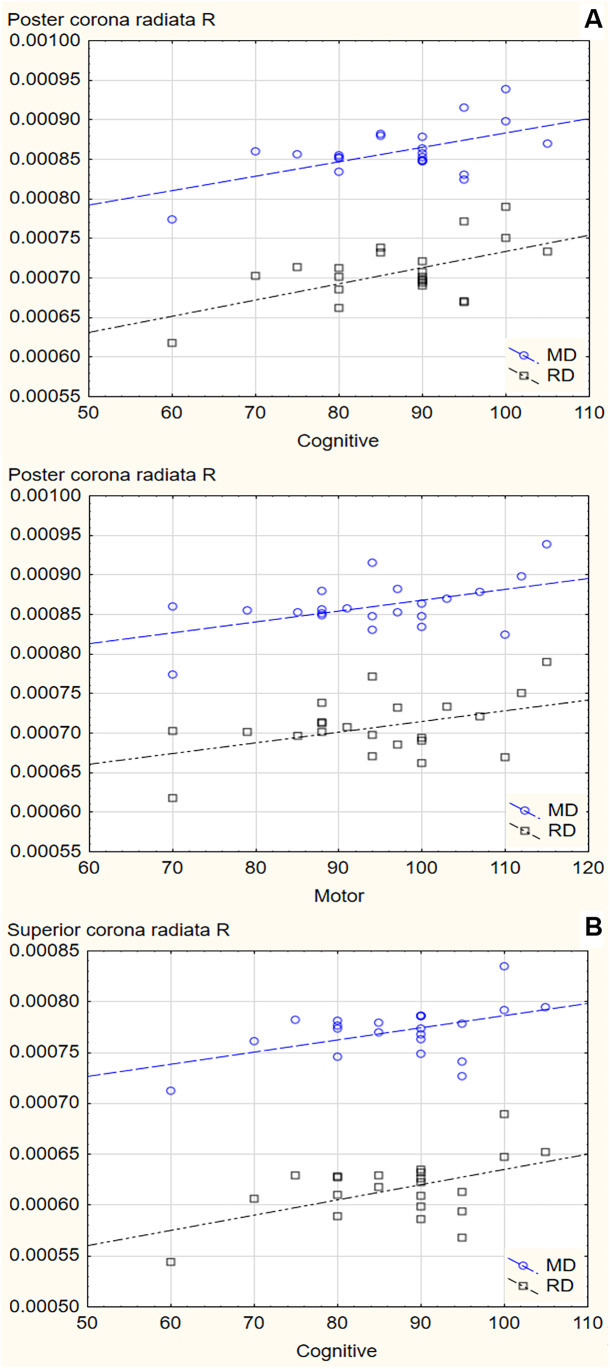
Associations of mean (MD) and radial (RD) diffusivity in the corona radiata with neurodevelopment at age 2-3 years within the group of children with prenatal depression exposure. A. Association of MD and RD in the right posterior corona radiata with cognitive and motor function. B. Association of MD and RD in the right superior corona radiata with cognitive function

Decreased MD and RD in the right posterior and superior corona radiata were associated with worse cognitive and motor function at age 2-3 years within the PDE group with large effect sizes (Table 3, Figure 3). There was no significant association within controls. There were no associations of white matter integrity with internalizing and externalizing behavior at this age.

**Table 3:** Associations of mean (MD) and radial diffusion (RD) in the right corona radiata with neurodevelopment in children with prenatal depression exposure at age 2-3 years

Sub-region	Bayley domain	Effect	b	Partial eta^2^	p
Posterior	Cognitive	MD	0.53	0.3291	0.016
		RD	0.52	0.3600	0.011
	Motor	MD	0.57	0.2724	0.032
		RD	0.50	0.2395	0.046
Superior	Cognitive	MD	0.55	0.3494	0.012
		RD	0.55	0.3546	0.012

Partial eta^2^ values were >0.1379 representing large effect sizes.

Models controlled for sex, age, alcohol use and tobacco smoking.

## Discussion

Our findings in 2-3-year-olds in this high-risk South African birth cohort, support the emerging literature reporting putative widespread effects of PDE on white matter microstructural integrity in young children. Although we did not detect group differences at neonatal age, we describe changes at 2-3 years associated with PDE in major association and projection tracts connecting cortical and subcortical regions including the sagittal stratum, superior fronto-occipital fasciculus, and corona radiata, and the posterior region of the cingulum which extends to the hippocampus of the limbic system. We further describe associations of these white matter alterations with cognitive development at this age.

Our findings suggest that the sagittal stratum may play a key role with regard to the impact of maternal depression on early child neurodevelopment. Group effects in 2-3-year-olds were large compared to group differences in other tracts, and controlling for substance use did not alter effects. The sagittal stratum is a large intricate white matter bundle that relays major tracts such as the inferior frontal-occipital fasciculus, and superior and inferior longitudinal fasciculi, between cortical and subcortical regions (Jellison et al., 2004). We observed differences in FA, MD and RD in PDE compared to controls in this region, a difference in RD suggesting altered myelination. Given the importance of the sagittal stratum in terms of connections, alteration of white matter integrity in this tract bundle may have widespread implications for effective integration of numerous functions including emotional processing, regulation of mental distress, sensory and motor function (Hinton et al., 2019; Juttukonda et al., 2019; Patel et al., 2018; Poletti et al., 2020). One prior study in neonates found sex-by-symptom interaction effects for FA in the sagittal stratum for a composite of prenatal depression and anxiety (Dean et al., 2018). The involvement of the sagittal stratum in adolescents and adults with major depression has been previously described (Henderson et al., 2013; Hermesdorf et al., 2017; Kieseppä et al., 2010; Ota et al., 2015). Further, studies in depressed middle-aged adults have also described altered FA, MD, RD and AD in the sagittal stratum (Kieseppä et al., 2010; Korgaonkar et al., 2011; Ota et al., 2015).

We also noted altered FA and RD in the superior fronto-occipital fasciculus, the latter parameter having lost significance when controlling for substance use. There is some support for altered MD and RD in tracts originating from frontal regions including the inferior fronto-occipital fasciculus in 2-5-year-old children with a history of PDE (Lebel et al., 2016). Since the superior frontal-occipital fasciculus had altered white matter integrity in adult mega studies of major depressive disorder (van Velzen et al., 2020), this tract may well be of interest across the lifespan following PDE. The superior fronto-occipital fasciculus extends to frontal and parietal lobes, and medially to the thalamus (Wakana et al., 2004). The study by Dean and colleagues (Dean et al., 2018) in neonates also reported altered microstructure in the posterior thalamic radiation, and in white matter adjacent to the hippocampus; consistent with our findings at 2-3 years of alterations in the posterior thalamic radiation and hippocampal portion of the cingulum.

Further, the posterior and superior corona radiata had altered white matter integrity in our cohort at 2-3 years. This is partly consistent with previous studies. Lebel and colleagues found in 2-5-year olds with PDE, associations of postpartum depression with diffusion in tracts of the superior frontal cortex including the anterior portion of the corona radiata (Lebel et al., 2016); while studies in neonates with prenatal exposure to depression and anxiety found altered MD, RD and AD across the corona radiata (Dean et al., 2018). These tract regions also extend to the posterior thalamic radiation, while the larger corona radiata connect most cortical regions via a mixture of projection, association and callosal tracts (Jellison et al., 2004; Wakana et al., 2004).

It is unclear why we did not observe associations of PDE at neonatal age in our cohort as inconsistent with some previous findings. We had some overlap in the children scanned as neonates and at 2-3 years. This may be explained by challenges common to neuroimaging of young children including different neurodevelopmental stages, practical difficulties, movement and other technical artefacts resulting in smaller sample size (Barkovich et al., 2019; Raschle et al., 2012; Thieba et al., 2018).

Regarding associations with development in our group of 2-3-year-olds with PDE, this is the first time to our knowledge, that altered white matter integrity in PDE has been associated with worse cognitive function at this early age. It is notable that we observed associations in the corona radiata, considering evidence of it being affected following PDE in this as well as other paediatric cohorts, and in adults with major depression. In our cohort, lower RD, suggesting altered myelination, and lower MD, both associated with poorer cognitive and motor function in 2-3-year-olds following PDE. In a separate analysis in the larger DCHS cohort, antenatal maternal depression associated with poorer cognitive function at 2-3 years (Donald et al., 2019). This is consistent with a recent longitudinal study of 2679 children exposed to pre- and postpartum maternal depression that described cognitive and motor delays at age 2 years (Chorbadjian et al., 2020). Another study of 223 infants aged 18 months found poorer cognition after PDE independent of postnatal depression (Koutra et al., 2013). Further, white matter integrity of the corona radiata, described as having a central role in emotion regulation, was particularly affected in adults with major depression (van Velzen et al., 2020).

We did not find white matter alterations to associate with internalizing and externalizing symptoms at 24 months in this study. This is inconsistent with a small literature addressing this issue. One study in neonates with PDE, using clustering coefficients of individual structures derived from DTI data, found that this coefficient of the right amygdala predicted later internalizing and externalizing behavior at 24 months (Wee et al., 2017). Other researchers have found, associations of frontal functional connectivity with internalizing and externalizing behavior at 24 months following PDE (Soe et al., 2016). In turn, altered structural connectivity in prefrontal-limbic pathways mediated externalizing behavior following PDE in 2-6-year-old children (Hay et al., 2020). The CBCL may not be a reliable measure in our setting or at this very young age when parent reports of behavior challenges tend to be less stable.

The pattern in this cohort of widespread associations of PDE with decreased white matter integrity is notable. Though studying the effects of maternal depression on subsequent neurodevelopment of offspring, our findings are consistent with an adult literature on depression, and widespread alterations in white matter integrity (Davis et al., 2019; van Velzen et al., 2020). These continuities across the life span are consistent with the Developmental Origins of Health and Disease (DOHaD) model of intergenerational transfer of biological alterations through epigenetic mechanisms, including for maternal distress especially in LMIC settings where the risk for development of major depression is high (Monk et al., 2019). One study of neonates exposed to major depression prenatally, found associations of depression with modified control of synaptic plasticity in cortical-limbic regions of neonates (Qiu et al., 2017). These and other complex neurobiological changes have been associated with cognitive, emotional and behavioral outcomes in children following PDE (Gelaye et al., 2016; Gentile, 2017; Herba et al., 2016). In support as described above, we found altered white matter integrity in the corona radiata, in this cohort with poorer cognitive and motor performance at age 2-3 years.

A number of limitations deserve emphasis. First, we did not determine whether postnatal depression further contributed to the association of PDE with white matter integrity, as few mothers (n=7) in our sample had persistent depression. Second, although we controlled for key confounders known to affect white matter microstructure, we cannot rule out the potential for residual confounding from HIV status and unmeasured factors. Thirdly, having converted our sample’s representative template to standard FA and MNI space during image processing may have de-optimized registration. However, our findings in the brains of 2-3-year-olds coincided with findings in the field. Fourthly, due to the exploratory approach to our analysis most findings did not survive FDR correction for multiple comparisons and effect sizes were reported. Our results should therefore be interpreted with caution and be replicated in further studies.

## Conclusions

Our findings of white matter alterations in 2-3-year-olds coincide with previous findings in children with PDE and adults with major depression. To our knowledge this is the first evidence for associations of altered white matter integrity with neurodevelopment in this age group of children with PDE. As PDE may have potentially long-term impact, it is important that maternal depression be identified and addressed early. This is especially pressing in LMIC settings such as our study setting, where rates of depression are much higher than in the general population (Mokwena & Masike, 2020), and where psychological interventions may be useful (Gajaria & Ravindran, 2018). The rate of depression in our larger DCHS cohort of 28% coincide with the higher rates in LMIC. Given these findings in young children of mothers with depression in pregnancy, that show such high consistency with studies of the effect of depression on the adult brain, there is the possible role of the brain as potential mediator for the intergenerational risk for depression. Further study is needed to assess longitudinal changes in white matter microstructure, along with cognitive and behavioral outcomes, and the mechanisms underlying associations with PDE.

## Data Availability

Data of this study are available from the authors upon reasonable request as per cohort guidelines.
